# Effect of *Aloysia citrodora* Essential Oil on Biochemicals, Antioxidant Characteristics, and Shelf Life of Strawberry Fruit during Storage

**DOI:** 10.3390/metabo11050256

**Published:** 2021-04-21

**Authors:** Habib Shirzad, Abolfazl Alirezalu, Kazem Alirezalu, Milad Yaghoubi, Bahareh Ghorbani, Mirian Pateiro, José M. Lorenzo

**Affiliations:** 1 Department of Horticultural Sciences, Faculty of Agriculture, Urmia University, Urmia P.O. Box 165-5715944931, Iran; h.shirzad@urmia.ac.ir (H.S.); a.alirezalu@urmia.ac.ir (A.A.); ghorbani.bahareh@ymail.com (B.G.); 2 Department of Food Science and Technology, Ahar Faculty of Agriculture and Natural Resources, University of Tabriz, Tabriz 51666, Iran; kazem.alirezalu@tabrizu.ac.ir; 3 Department of Food Science and Technology, Faculty of Agriculture, University of Tabriz, Tabriz 51666, Iran; m.yaghoubi97@ms.tabrizu.ac.ir; 4 Centro Tecnológico de la Carne de Galicia, Parque Tecnológico de Galicia, rúa Galicia No. 4, San Cibrao das Viñas, 32900 Ourense, Spain; mirianpateiro@ceteca.net; 5 Área de Tecnología de los Alimentos, Facultad de Ciencias de Ourense, Universidad de Vigo, 32004 Ourense, Spain

**Keywords:** *Aloysia citrodora*, strawberry, essential oil, antioxidant, shelf life

## Abstract

Strawberry fruits are highly susceptible to cold burning, resulting in low storage periods at low temperatures. Plant extracts or essential oils (EOs) can potentially be used as preservatives in fruits throughout the refrigerated period. In the present study, the biochemicals, antioxidant characteristics, and shelf life of treated strawberries with *Aloysia citrodora* essential oil (ACEOs) were evaluated during keeping time. The treatments were produced as follows: T1, control; T2, 250 ppm ACEOs; T3, 500 ppm ACEOs; and T4, 750 ppm ACEOs. Total soluble solids (TSS), weight loss, titratable acidity (TA), antioxidant activity (DPPH assay), total phenolic (TPC), flavonoid and anthocyanin contents (TFC), and enzymes activity (peroxidase and ascorbate peroxidase) were evaluated during the refrigerated period (5 °C with relative humidity of 85–90% for 20 days). The results revealed that weight loss and TA were reduced in all treatments during storage, being that the rates were lower in samples treated with ACEOs. TPC, TFC, TSS, antioxidant, and enzymes activity were higher in treated fruits than control.

## 1. Introduction

Strawberry (*Fragaria* × *ananassa* Duch, from Rosaceae family) is one of the most perishable fruits, which could be related to its high respiration, high water content, high metabolic activity, and susceptibility to microbial contamination. The quality of the fruit depends on the appearance, texture, aroma, taste, and nutritional values [1]. The strawberry is a rich source of ascorbic acid and anthocyanin, which not only give antioxidant properties to the strawberries, but also many health benefits, such as enhancing the body’s immune system and reducing the risk of developing cancers [2,3].

The applications of artificial antimicrobials for increasing the fruit’s shelf life are linked to toxicity effects, which has led to the search for healthier options. Therefore, the recent use of natural sources in fruits to control quality properties is known as one of the more efficient techniques [4,5]. In this regard, the application of essential oils (EOs) could lead to delaying fruit spoilage, maintaining the quality, and increasing the shelf life [6]. EOs have potential antifungal properties and are used as disinfectants, which would allow them to reduce early fruit deterioration and increase the fruit’s post-harvest life [7]. In recent decades, many efforts have been made to increase the shelf life of the strawberry fruit and maintain its nutritional values [8]. Maintenance of foods using methods such as controlled atmosphere [9] and the use of natural compounds [10] have been reported. One of the suitable and operational methods for increasing shelf life and maintaining quality attributes is the application of edible coatings based on natural essential oils [11]. Chitosan coating enriched with lemon EO has been used to protect strawberries from gray mold [12]. In addition, the combination of thyme EO with edible coatings is an effective way to minimize the deterioration of fruit by controlling microbiological growth, and also directly leads to improving the fruit quality [13].

The *Aloysia citrodora* plant contains neral, 1,8-cineole, geraniol, limonene, tannin, phenolic acids, flavonoids, and alkaloids [14]. Many studies have been conducted on the phytochemicals and antioxidant properties of the *Aloysia citrodora* plant [15], which have confirmed its antioxidant properties [16]. In addition, the protection effect of the *Aloysia citrodora* plant has been proven [15]. The activities of catalase, glutathione peroxidase, and glutathione reductase enzymes in damaged cells have increased. Post-harvest damages of strawberry fruits, at cold storage, including fungal pathogens, water and biochemical loss, and softening resulted in a low shelf life of the strawberries (approximately 5 days). Therefore, the aim of the present work was to extend the shelf life of strawberries by using non-chemical post-harvest treatment. Particularly, the impacts of different levels of *A. citrodora* EOs throughout post-harvest cold storage on enzymatic activities, antioxidant capacity, and biochemical constituents in strawberries were assessed. The obtained results can provide natural and Gras resources for the food industry and post-harvest technology.

## 2. Results and Discussion

### 2.1. Weight Loss

**Figure 1:**
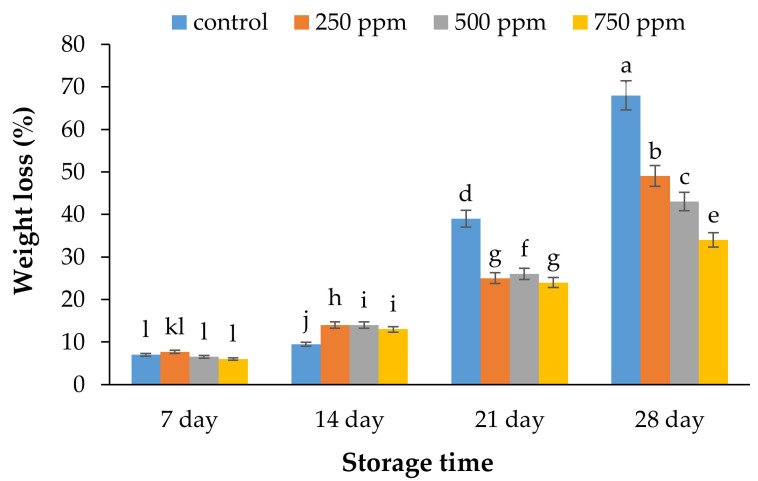
Effect of ACEOs on the weight loss of strawberries during storage. Different letters indicate a significant difference (*p* < 0.01) between samples.

The weight loss and water loss of fruit samples were both decreased throughout the keeping period, which may have been caused by evaporation. Based on obtained results, the samples treated with ACEOs showed a lower weight loss compared to the control. The samples treated with 750 ppm ACEOs displayed the lowest percentage of weight loss compared to other treatments during the storage period (Figure 1). The results also indicated that treated samples with 750 ppm ACEOs displayed 50% lower weight loss than those observed in control samples. This demonstrated that several post-harvest treatments such as chemical treatments [17], coating [18], and physical treatments [19] can reduce cold burning, thus reducing susceptibility to fungal and bacterial contamination.

### 2.2. Chemical Characteristics (Titratable Acidity and Total Soluble Solid)

**Figure 2:**
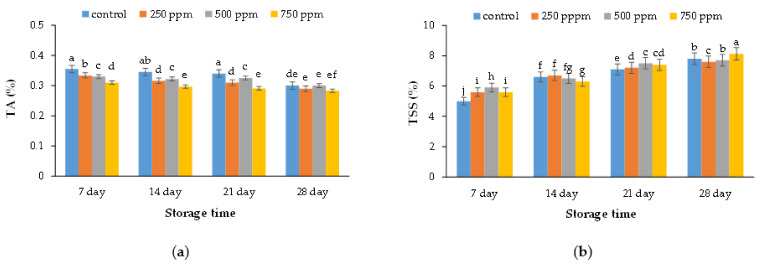
Effect of ACEOs on the quality attributes TA (**a**) and TSS (**b**) of strawberries during storage. Different letters indicate a significant difference (*p* < 0.01) between samples.

The results indicated that the TSS of control samples was lower than that of treated samples. Treated samples with 750 ppm ACEOs had the highest dissolved solids content (Figure 2a). The amount of total carbohydrates was one of the most important factors in increasing the texture resistance of the fruit to stress, since conversion of starch into reducing sugars is a positive factor during stress. In addition, the fact that most organic acids are secondary metabolites that are formed as a result of a citric acid cycle, and are used during respiration because acidity increases with raising storage duration, confirms the results of the present study [20].

Based on the obtained results, the control and treated samples with 750 ppm ACEOs displayed the highest and lowest amount of TA, respectively (Figure 2b). The results revealed that the acid levels decreased in strawberry samples during the keeping period (particularly in low-temperature rooms), which may be attributed to acids breaking down into sugar throughout fruit respiration. On the other hand, enzymatic activities in fruit juice during the keeping period could be another reason for acidity reduction [21]. The organic acids consumed during respiration would support normal activities in fruit samples during storage time [22]. However, enzymatic reactions and respiration could be decreased by edible coatings [23]. Edible coatings were capable of creating a modified atmosphere around fruit samples and maintaining CO_2_ at higher amounts; therefore, EOs decreased the consumption of organic acids by reduction in respiration and production of ethylene [24].

The organic acids accumulation during the fruit ripening process played an important role in the carboxylic acid glycolysis cycle. The fluctuation in organic acids led to changes in acidity; however, increasing in one parameter led to decreasing in other one. Therefore, acidity in orange fruits was affected by various types of acids such as citric acid, malic acid, benzoic acid, tartaric acid, and oxalic acid [25]. Carbohydrates and organic acids in the fruit could potentially influence taste and flavor attributes. However, most of the organic acids were secondary metabolites of the citric acid cycle during fruit breathing; therefore, acidity increased throughout the storage period. These results are paralleled with previous results reported by Burdurlu et al. [26] and Shao et al. [27].

There is limited research on the organoleptic attributes of treated fruits with EOs. Neri et al. [28] showed that added (E)-hex-2-enal in nectarine and peach fruits as “green” (leafy) resulted in off-odors. However, off-odors declined throughout ripening, and nothing was perceived after ripening. In contrast, applied carvacrol or citral had no bad effects on the odor of nectarine and peach fruits. According to results reported by Aloui et al. [12], there were no off-odors or off-flavors in dates treated with citrus EOs. Serrano et al. [29] also evaluated the effects of thymol and eugenol on cherries and reported no organoleptic effects. Prasad and Stadelbacher [30] indicated that acetaldehyde in low concentration (1%) did not reveal any off-flavor in strawberries, while a higher concentration of acetaldehyde (4%) resulted in off-flavor.

According to reviewed articles, a wide range EOs do not leave bad effects on intact fruit odors and flavors at low levels, particularly when stored fruits finish their ripening process. However, there is variation among cultivars and fruit species, and each commodity has to be tested separately. For instance, Neri et al. [28] indicated that pome fruits were less sensitive against (E)-hex-2-enal injury compared to stone fruits, which may be attributed to higher absorption of (E)-hex-2-enal by pome fruits.

### 2.3. Antioxidant Activity by DPPH Assay

**Figure 3:**
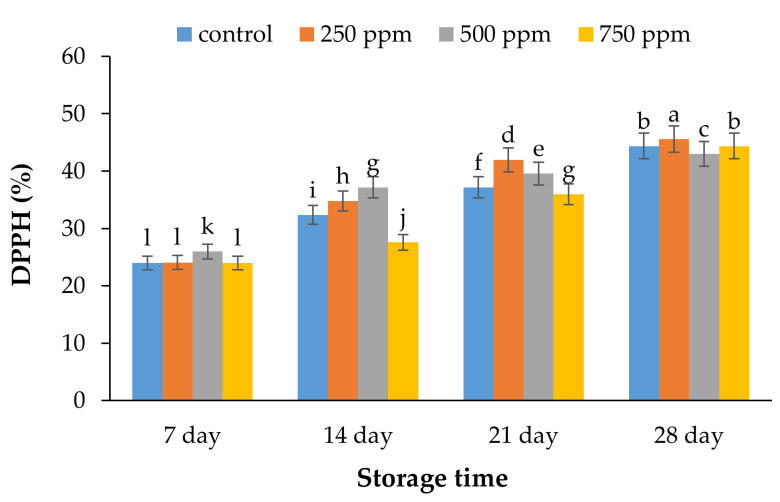
Effect of ACEOs on the antioxidant activity of strawberries during storage. Different letters indicate a significant difference (*p* < 0.01) between samples.

The outcomes of DPPH values indicated that antioxidant capacity of the samples was at its highest content at the end of the keeping time. The antioxidant properties of the samples displayed that 250 ppm AFEOs had the highest antioxidant activity compared to other treatments (Figure 3). The results indicated that the use of *A. citrodora* EOs in edible coatings, by decreasing the rates of oxidation reactions, could reduce the damage of reactive oxygen species. However, by increasing the concentration of EOs, the antioxidant activities of coating increased. Antioxidant activity was increased throughout keeping time in all samples, and the rate of this activity was higher in treated samples compared to the control. The antioxidant activities of fruit samples are potentially related to the content of flavonoids and phenols, which are presented highly in plant extracts and EOs [31]. In recent decades, the application of medicinal plant extracts and EOs has become known as a new and safe method to improve the quality stability and the shelf life of the samples [32,33]. According to Alirezalu et al. [34] and Shameh et al. [35], the findings of the antioxidant capacity of strawberries were related to phenolic compounds such as tannin, and TFC. Synowiec et al. [36] evaluated the effects of *Ocimum basilicum* extract on apples. The authors demonstrated that the antioxidant capacity of the samples was effectively increased throughout the keeping period. According to results reported by Sellamuthu et al. [37], the herbal extracts and EOs improved berry fruits’ antioxidant capacity, quality stability, and shelf life.

### 2.4. Total Phenolic Content (TPC)

**Figure 4:**
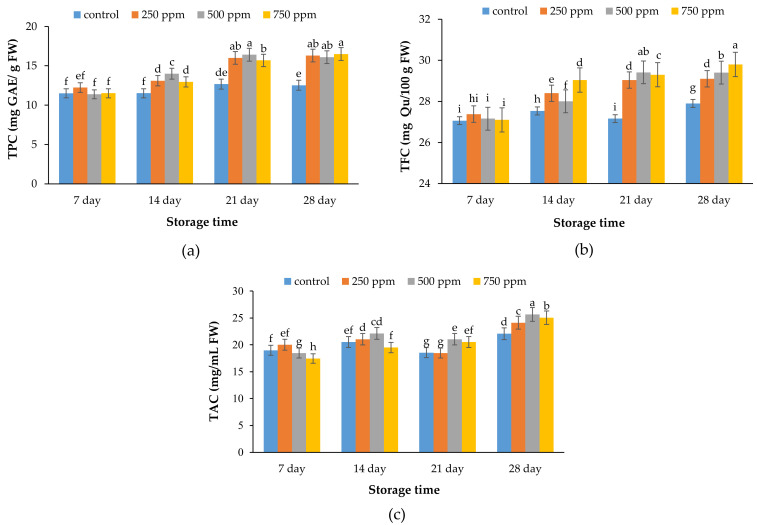
Effect of ACEOs on (**a**) total phenolic content, (**b**) total flavonoid content, and (**c**) total anthocyanin content of the strawberries during storage. Different letters indicate a significant difference (*p* < 0.01) between samples.

Fruit samples treated with 250 and 500 ppm ACEOs displayed the highest amounts of total phenolic compounds (Figure 4a). In contrast, higher TPC of treated samples with 500 ppm ACEOs as compared to 750 ppm ACEOs at days 14 and 21 might be attributed to the toxic impacts of higher EO levels on the cells, resulting in breakdown of the cellular structure, which could be related to aging and the enhanced activity of the polyphenol oxidase enzyme [38]. Plant tissues are a rich source of phenolic compounds, including flavonoids, tannins, hydroxycinnamic esters, and lignin [39]. The higher amounts of phenolic compounds in plant sources might be the main reason for the higher total phenolic compounds in treated samples with ACEOs compared to the control. As expected, samples treated with 750 ppm ACEOs had the highest phenol levels. Phenolic compounds in plant materials are highly susceptible to environmental and biological stresses [40]. The phenolic compounds play an important role in reducing or inhibiting lipid autoxidation, eliminating free oxygen radicals, or decomposing peroxides [41]. The results of this work indicated that treated samples with higher concentrations of ACEOs displayed higher amounts of TPC, which paralleled results reported by Yang et al. [42] and Wang and Xu [43] on blueberry and strawberry, respectively.

Phenolic compounds, as secondary metabolites of plants, have potential antimicrobial properties [44]. Stimulation of phenolic compounds in treated strawberries by ACEOs may be the main reason for low decay occurrence in the samples. According to previous results, plants’ EOs are a new and safe method to control post-harvest pathogens of berry fruits. On the other hand, these compounds improve the quality attributes and shelf life of berries [37]. The results of the present work indicated that treated strawberries with ACEOs displayed significantly higher amounts of TPC compared to the control at day 28.

### 2.5. Total Flavonoid Content (TFC)

The results of total flavonoid content are displayed in Figure 4b. The results indicated that samples treated with 750 ppm ACEOs had significantly higher TFC compared to other groups. Previous results may be attributed to the fact that plant extracts or EOs are a rich source of phenolic and flavonoid compounds. In this way, higher amounts of flavonoids (as potential antioxidant ingredients) in treated samples could increase the shelf life of strawberries, compared to the control.

Total phenolic content, particularly flavonoids (TFC) with potential antioxidant activities, are extremely beneficial to consumers. These components can deactivate free radicals in plants, which may be caused by hydroxyl groups [45]. Ripening of the fruits throughout the storage could be the main reason for the increase in flavonoids in the present work [21]. The control samples displayed a lower content of flavonoids than the coated samples. The types of fruits, as well as composition index, could affect the amount of flavonoids [45].

The content and types of flavonoids are one of the most important ingredients in plants for controlling stress tolerance [46], and these are increased under stress [20]. Therefore, flavonoids play an important role in control of plant stress types due to their antioxidant properties and their specific structure. In fact, strawberries treated with 750 ppm ACEOs showed significantly higher amounts of TFC compared to other groups at the end of the storage period.

### 2.6. Total Anthocyanin Content (TAC)

The results of the total anthocyanin content are illustrated in Figure 4c. The color of the strawberry fruit is derived from anthocyanin content. In this regard, da Silva et al. [47] found that more than 25 anthocyanins have been reported in different cultivars of strawberries, with pelargonidin 3-O-glucoside, pelargonidin 3-O-rutinoside, and cyanidin 3-O-glucoside being the main components. TAC of all samples decreased gradually during storage, which may be caused by oxidation reactions.

### 2.7. Peroxidase Enzyme Activity

**Figure 5:**
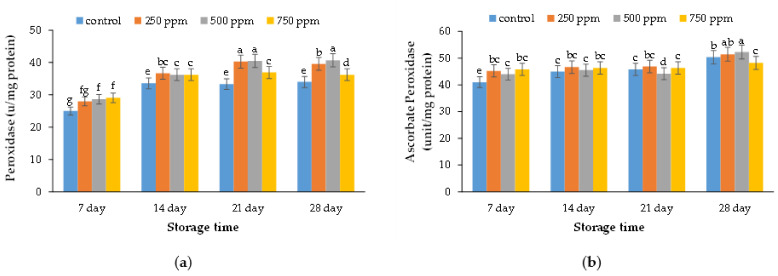
Effect of ACEOs on the (**a**) peroxidase and (**b**) ascorbate peroxidase enzyme activity of strawberries during storage. Different letters indicate a significant difference (*p* < 0.01) between samples.

The results of the peroxidase enzyme activity of strawberries are presented in Figure 5a. The results indicated that treated strawberries with 500 ppm ACEOs had the highest levels of peroxidase enzyme activity. Peroxidases play a vital role in metabolic steps of the cell, such as auxin catabolism, lignin synthesis, and suberin [48]. Peroxidases in the presence of phenolic compounds and ascorbic acid can act as an effective inhibitor against apoplast and vacuole [49]. Therefore, the peroxidase with potential antioxidant properties could extend the shelf life of products [3].

### 2.8. Ascorbate Peroxidase Enzyme Activity

The results of the ascorbate peroxidase enzyme activity of strawberries are illustrated in Figure 5b. The results showed that the highest level of peroxidase enzyme was observed in strawberries treated with 500 ppm ACEOs. Ascorbate peroxidase enzymes activity could prevent plants from being damaged by activated oxygen species when exposed to severe stress. Afshar Mohammadian et al. [50] proved that the cold resistance of two olive cultivars could be related to the total protein content, which was higher in sensitive olive cultivars. In addition, during cold stress the defensive enzymes activity against oxidative stress was increased; while after the cold stress, the protein storage of the plant increased to return to the normal state [51].

## 3. Materials and Methods

### 3.1. Fruits

The test cultivar utilized in the present work was Albion (*Fragaria* × *ananassa* Duch. cv. Albion). The strawberries were taken at commercial maturity stage from a commercial greenhouse in Urmia, West Azerbaijan province, Iran. Sampling was carried out early in the morning. The fruit was selected for the similarity of size, maturity, and color, and the damaged and unshaped fruit samples were removed. The samples were then transferred in a fridge of 4 ± 1 °C to the Department of Horticulture Science of Faculty of Agriculture of Urmia University (Urmia, Iran).

### 3.2. Preparation of ACEOs and Treatments

In order to extract *Aloysia citrodora* essential oil, dried leaves were used for water distillation using a Clevenger apparatus. After preparing different concentrations of ACEOs (0, 250, 500, and 750 ppm), and due to their lipophilic properties and insolubility in distilled water, samples were mixed with Tween 80 (0.5%, *v*/*v*). Then, the samples (60 g) were dipped in the ACEOs for 5 minutes. The fruits were put into sterilized packaging. Finally, all samples were transferred to a refrigerator (5 °C, with relative humidity of 85–90%) and analyzed at 1, 7, 14, 21, and 28 days of keeping time.

### 3.3. Weight Loss

For determination of the samples’ weight loss, 15 samples were selected and weighed. The percentage of weight loss was calculated as follows [52]:

(1) $$\mathrm{Weight}\;\mathrm{Loss}\;\left(\%\right)=\frac{\mathrm{Primary}\;\mathrm{weight}-\mathrm{secondary}\;\mathrm{weight}}{\mathrm{Primary}\;\mathrm{weight}}\times 100$$

### 3.4. Chemical Characteristics (Titratable Acidity and Total Soluble Solid)

Total acidity was calculated by the titration method using sodium hydroxide (0.1 N) in terms of citric acid. In other words, it was titrated with soda solution (0.1 N) to reach pH 8.3. After applying, the value of the used soda was introduced in the following formula, and the acidity was calculated based on g of citric acid in 2 L of fruit extract and then converted to a percent [53]:

(2) $$\mathrm{TA}=\frac{100\times M\times N\times V}{S\times n}$$

where TA = acidity value based on g of citric acid in 2 L sample extract, M = molecular weight of the dominant acid, n = dominant acid capacity, V = volume of the used soda, S = amount of the extract used, and N = normality of the used soda.

A manual refractometer (ATAGO, Tokyo, Japan) was utilized for measurement of total soluble solid (TSS) after calibration with distilled water [54].

### 3.5. Antioxidant Activity by DPPH

Firstly, the juice of the samples was extracted by pressure [55] and centrifuged at 11,000× *g* at 4 °C for 15 min. The obtained juice was utilized for evaluation of DPPH, TPC, and TFC.

The total antioxidant properties of the samples were evaluated by 2,2-diphenyl-1-picrylhydrazyl-hydrate (DPPH) method. For this purpose, 2000 μL of the DPPH (pre-prepared) solution was poured into the sterilized test tubes. Then, a specific amount of the fruit extract of each of the samples was added, and the resulting solution was shaken at room temperature and placed in the dark for 30 min. The value of the absorbance of the resulting solution was read by a spectrophotometer at a wavelength of 517 nm. The above method was also used to prepare the control, but instead of the extract, 80% methanol was used and calculated according to the following formula [56]:

(3) $$\mathrm{RSA}=\frac{\left(\mathrm{Abs}\;\mathrm{control}\right)t=30\;\min-\left(\mathrm{Abs}\;\mathrm{sample}\right)t=30\;\min}{\left(\mathrm{Abs}\;\mathrm{control}\right)t=30\;\min}\times 100$$

### 3.6. Total Phenolic Content (TPC)

TPC was determined using Folin–Ciocalteu reagent according to the method of Ali-rezalu et al. [4]. A total of 30 μL concentrated extract was poured into the test tube, and 90 μL distilled water was added. Then, 600 μL of 10% Folin was added, and after 10 min, 480 μL of 7.5% sodium carbonate was added, and after placing it for 30 min in the dark at room temperature, a spectrophotometer (UV-1800, Shimatzu Corporation, Kyoto, Japan) was used to read the absorbance at 760 nm. Gallic acid was used as a standard. The total phenol content of extracts was expressed as mg of gallic acid equivalent (GAE)/g FW of the sample.

### 3.7. Total Flavonoid Content (TFC)

To measure the total flavonoid content, 500 µL of the prepared extract was poured into the test tube, and 150 µL of 5% sodium nitrite was added. After 5 min, 300 µL of 10% aluminum chloride was added, and again, after 5–10 min, 1000 µL of 1% NaOH was mixed into the resulting solution and was brought to a volume of 5 mL with the deionized water. The absorbance of the resulting mixture was read at 380 nm, compared to the control. Quercetin was used to draw the standard curve. The total flavonoid content of the total extracts was expressed as mg of quercetin equivalent (Qu)/100 g FW of the sample [57].

### 3.8. Total Anthocyanin Content (TAC)

The pH difference method was used to measure the total anthocyanin content. For this purpose, the fruit juice was centrifuged at 11,000× *g* for 15 min at 4 °C, and then, 100 µL of a supernatant was used. Two buffers were prepared with pH 1 and 4. Then, 2.5 mL of buffer 1 was poured into the test tube. Afterward, 100 μL of the extract was added to the solution poured into the test tube, and the absorbance at two wavelengths of 700 nm and 530 nm was read. Next, 2.5 mL of buffer 2 (pH 4.5) was poured into another test tube and 100 μL of the extract was added, and the absorbance at two wavelengths of 700 nm and 530 nm was read. Finally, the following formula was used to be calculated the total absorbance of each of the extracts [35]:

A = (A530 − A700) pH = 1 − (A530 − A700) pH = 4.5(4)

Total anthocyanin content was calculated according to the following formula:

(5) $$\mathrm{TAC}=\frac{A\times \mathrm{MW}\times V\times \mathrm{DF}\times 100}{\varepsilon \times 100}$$

where A = absorbance, MW = molecular weight, DF = dilution factor, and ε = molar absorbance. The results were expressed as mg of cyanidin-3-O-glucoside/mL of fruit juice.

### 3.9. Peroxidase Enzyme Activity

Peroxidase enzyme activity of the samples was evaluated according to Mohammadi et al. [58]. Briefly, 2 mL of tris buffer (100 μM, pH = 7.5), 300 μL of oxygen peroxide of 5 μm, and 200 μL guaiacol (10 μM) were mixed in an ice bath. Then, 50 μL of enzyme extracts was added. The curve of absorption changes was assessed by spectrophotometry at 425 nm. The peroxidase enzyme activity was expressed as u/mg protein of the sample.

### 3.10. Ascorbate Peroxidase Enzyme Activity

Ascorbate peroxidase was assayed from the decrease in absorbance at 290 nm as ascorbate was oxidized [59]. The samples were powdered in liquid nitrogen, and the enzymatic extract was extracted in Na_2_HPO_4_/KH_2_PO_4_ buffer at 50 mM and pH = 7. The extract was centrifuged at 10,000× *g* for 10 min. For the measurement of enzyme activity: 300 μL of sodium phosphate buffer (pH = 7, containing 0.2 EDTA mM), 200 μL of ascorbic acid (0.5 mM), 200 μL of Bovine Serum Albumin (BSA), 50 μL of enzyme extract, and 50 μL of hydrogen peroxide (250 mM) were mixed. Absorption changes were measured by spectrophotometry at 290 nm for 3 min. The enzyme activity was calculated using the extinction coefficient of ascorbic acid (mM^−1^ cm^−1^) according to unit/mg protein of the sample.

### 3.11. Statistical Analysis

This experiment was carried out based on a completely randomized design with 4 (concentrations of essential oils) × 4 (storage times) × 4 replications (each replication included 60 g fruits). One-way ANOVA was used for analyzing the data (SAS, version 9.1.3, SAS Institute Inc., Cary, NC, USA). The means of scores were compared using Duncan’s Multiple Range Test. Differences at *p* < 0.01 were considered significant.

## 4. Conclusions

The aim of the present work was to extend the shelf life of strawberries by using non-chemical post-harvest treatment. Treated strawberries with *Aloysia citrodora* EOs had less weight loss and higher biochemical contents, antioxidant properties, and enzyme activity compared to control. According to obtained results, the *Aloysia citrodora* EOs (particularly 500–750 ppm) could potentially improve shelf life, safety, and biochemical components retention by decreasing the decay rate in strawberries in cold storage. The outcomes of the present work provide new safe and Gras additives to the production of new products for the post-harvest and food industries, but further research is needed to obtain more details on *Aloysia citrodora* EOs.

## Data Availability

The data presented in this study are available on request from the corresponding authors. The data are not publicly available due to being part of a research project.
